# Metallothionein-1A (MT1A) Gene Variability May Play a Role in Female Frailty: A Preliminary Study

**DOI:** 10.3390/genes16010015

**Published:** 2024-12-26

**Authors:** Paolina Crocco, Francesco De Rango, Rossella La Grotta, Giuseppe Passarino, Giuseppina Rose, Serena Dato

**Affiliations:** Department of Biology, Ecology and Earth Sciences, University of Calabria, 87036 Rende, CS, Italy; paolina.crocco@unical.it (P.C.); francesco.derango@unical.it (F.D.R.); rossella.lagrotta@unical.it (R.L.G.); giuseppe.passarino@unical.it (G.P.); pina.rose@unical.it (G.R.)

**Keywords:** metallothionein, MT, zinc, frailty, sex differences, sex specificity, SNP, health status, aging

## Abstract

Background/Objectives: Frailty is a complex geriatric syndrome resulting in decreased physiological reserve. While genetics plays a role, the underlying mechanisms remain unsolved. Metallothioneins (MTs), metal-binding proteins with high affinity for zinc, an essential mineral for many physiological functions, are involved in processes including oxidative stress and inflammation. We investigated the impact of genetic variations in MTs on frailty. Methods: 448 subjects (235 females and 213 males, median age of 76 years) were categorized into three frailty groups (non-frail/pre-frail/frail), by hierarchical cluster analysis based on cognitive status (MMSE), functional capacity (ADL), and physical strength (HGS). Subjects were analyzed for selected SNPs in *MT1A*, *MT1B*, *MT2A*, and *MT3* genes by PCR-RFLP. Results: An association was found between the rs8052394-A/G (Lys51Arg) polymorphism in the *MT1A* gene and frailty in females both in binary (OR = 0.345, *p* = 0.037) and multinomial logistic regression (OR = 0.343, *p* = 0.036) corrected for age and sex, with carriers of the minor G-allele less likely to transition from non-frail to pre-frail status. Additionally, a significant association with albumin levels (beta = 0.231; *p* = 0.027) and a trend of association with CRP levels (beta = −1.563; *p* = 0.097) were observed for this SNP in non-frail females, both indicative of a low inflammatory status. However, Bonferroni correction for multiple SNPs and physiological parameters tested renders these results statistically non-significant. Conclusions: Although its associations do not survive Bonferroni correction, this exploratory study suggests a sex-specific influence of *MT1A* variability in frailty, likely affecting zinc availability, aligning with ongoing research on sex differences in frailty risk and progression. Larger studies are needed to validate these findings and clarify the mechanisms behind MTs’ variability in frailty progression.

## 1. Introduction

Frailty is a health condition closely linked to aging, very common among older adults with a prevalence ranging from 19% to 75.6% in individuals aged over 60 years, with age-, gender- and country-specific differences [1]. Frail people have limited physical reserves, making them more susceptible to illnesses, falls, or hospital stays. Among older adults, frailty often co-occurs with cardiovascular diseases, diabetes, and Alzheimer’s disease [2], forming a vicious cycle where these conditions can exacerbate each other. Notably, frailty is a more significant predictor of mortality than chronological age [3]. This multifaceted syndrome arises from the interplay of various biological, psychological, and social factors [4]. The overlap between frailty and age-related disorders underscores its complex origins. To develop effective interventions to delay frailty and its associated health declines, it is crucial to unravel the shared biological mechanisms underlying these overlapping conditions. Recent research has highlighted the genetic component of frailty, with heritability estimates ranging around 45% consistently among populations [5,6,7]. Genome-wide association studies (GWAS) and candidate gene studies have identified numerous genetic variants associated with frailty [8,9,10,11,12,13,14], confirming its polygenic nature. These genes are often linked to established hallmarks of aging and age-related diseases, including inflammation, insulin signaling, muscle function, and cellular homeostasis. However, these loci only explain a fraction of the heritable risk, estimated around 11% [13], suggesting that additional genetic factors remain to be discovered.

Metallothioneins (MTs) are a class of small, cysteine-rich metal-binding proteins found in most living organisms. In mammals, MTs primarily bind zinc, but can readily exchange it for copper or cadmium when exposed to excessive levels of these metals [15]. In humans, the MT family is divided into four subfamilies of genes (*MT1*, *MT2*, *MT3* and *MT4*) clustered on chromosome 16q12-22. *MT1* and *MT2* are ubiquitously expressed, while *MT3* is brain-specific, and *MT4* is primarily found in squamous cell epithelium [16]. MTs have been shown to participate in a diverse array of intracellular functions. These include the regulation and storage of intracellular zinc, the detoxification of heavy metals, the promotion of neuronal survival, and the control of cell growth and death. Additionally, MTs contribute to the defense of oxidative damage and inflammatory responses [15]. As such, a growing number of studies are revealing their potential involvement in the development of chronic diseases. For instance, studies from animal models have shown that MT1 and MT2 can prevent the development of obesity by interfering with insulin signaling [17,18]. MT1 and MT2 exhibit protective functions against cardiomyopathy [19] and intermittent hypoxia-induced renal injury [20]. They also protect against oxidative stress-induced neurodegeneration, particularly MT3 [21,22,23]. MTs appear to be also related to senescence and lifespan. While elevated MT levels have been observed in long-living organisms [18,24,25], the relationship between MTs and human aging seems to be more complex. Over the years, MT expression appears to follow an inverted U-shaped pattern with age-specific effects. In younger individuals, MTs act as antioxidants by protecting cells from oxidative stress, in older individuals elevated MT levels may contribute to zinc deficiency, impaired immune function, and increased susceptibility to age-related diseases, while the decreased levels found in healthy nonagenarians/centenarians suggest a possible positive selection for survival [25,26,27,28]. The role of MTs in age-related diseases has also been investigated by analyzing the effect of their genetic variability. Associations have been found for several traits [16,29,30]. Moreover, variation in *MT* genotypes has been associated with lifespan in humans [31]. Considering the above findings, we propose that genetic variations within the *MT1A*, *MT1B*, *MT2A* and *MT3* genes could confer susceptibility to frailty in older individuals. To explore this potential association, we undertook a preliminary cross-sectional study of 448 subjects aged 65–99 years, enrolled in Southern Italy and classified by their frailty levels.

## 2. Materials and Methods

### 2.1. Study Population

The analyzed sample included a total of 448 subjects (235 females, 213 males) aged between 65 and 99 years (average age: 78.4 years, median age: 76 years). All subjects were born in Calabria (Southern Italy), and their ancestors were ascertained up to the third generation. Samples were collected as part of numerous and appropriate recruitment campaigns conducted to monitor the quality of aging throughout the Calabria region. Socio-demographic characteristics and health status, including medical history and medication use, were acquired for each participant through a standardized questionnaire, administered during a medical visit and clinical examination. Ethical approval for this study was granted by the local Ethics Committee “Comitato Etico Regione Calabria-Sezione Area Nord” on 31 October 2017 (code n. 25/2017). All participants gave written informed consent.

### 2.2. Measurements

A standardized questionnaire was used to gather information on social, demographic, and clinical status during interviews with qualified researchers. The multidimensional evaluations encompassed tests on cognitive status, functional abilities and physical performance, comprising those used for classifying the subjects according to their frailty status, as established by Montesanto et al., 2010 [32]. In brief, according to this approach, everyone can be classified with respect to his/her frailty level, determined by applying a hierarchical cluster analysis (HCA) on specific geriatric parameters, including the Mini Mental State Examination (MMSE), to test cognitive status, Activity of Daily Living (ADL) and Hand Grip (HGS) strength as measurements of functional activity.

#### 2.2.1. Cognitive Performance

The Mini Mental State Examination (MMSE) was used to assess cognitive function, evaluating orientation, episodic memory, attention, language, and constructive functions [33], whose scores ranged from 1 to 30, and a score < 24 was used to diagnose cognitive impairment. For the analysis, MMSE scores were normalized for age and educational status, as suggested by Grigoletto [34].

#### 2.2.2. Functional Activity

The Activities of Daily Living (ADL) test was used to estimate disability [35]. In brief, the result of the test is a score which comprises several activities (bathing, dressing, toileting, transferring from bed to chair, and feeding) in which the participant is dependent or independent at the time of the visit. For the analysis, ADL scores were dichotomized by assigning one to the non-independent subject in all five items and zero otherwise.

#### 2.2.3. Physical Performance

Hand grip strength (HGS) was used as a measure of physical fitness and was carried out by using a handheld dynamometer (SMEDLEY’s dynamometer TTM) while the subject was sitting with the arm close to his/her body. The test was repeated three times with the stronger hand and the maximum of these values was considered.

#### 2.2.4. Hematological Parameters

Samples from the venous blood were withdrawn after an overnight fast of 12 h in the morning. Biochemical measurements reported in Table 1 were performed at the Italian National Research Center on Aging (Cosenza) using standard protocols.

### 2.3. Genotyping

DNA was extracted from whole blood according to standardized procedures. Seven candidate genetic variants in four MT genes [*MT1A* (rs11076161, rs8052394, rs11640851), *MT1B* (rs964372), *MT2A* (rs1610216, rs10636), *MT3* (rs45570941)] were selected from previous studies reporting significant genetic associations with age-related phenotypes/diseases. For SNP’s genotyping, PCR amplification and restriction fragment length polymorphism analysis (PCR-RFLP) were carried out. Basic characteristics of SNPs and restriction enzymes are reported in Table S1. PCRs of 35 cycles were performed on a GeneAmp9700 thermal cycler (Perkin-Elmer Cetus, Norwalk, CT, USA). Primers were synthesized by Euroclone (Milan, Italy). PCR reaction conditions, primer sequences and RFLP condition can be detailed under request to the corresponding author.

### 2.4. Statistical Methodology

For each SNP, departure from the Hardy–Weinberg equilibrium was assessed in controls by χ^2^ test. Logistic and multinomial regression models were used to estimate the influence of genetic variability on frailty status, considering a dominant model of inheritance for all the analyzed variants and using age and gender as covariates. Association results were reported as estimation of the odds ratios (ORs), their 95% confidence intervals (CIs), and relative *p*-values. Analysis of covariance (ANCOVA) followed by post hoc tests was used to compare the mean differences in biochemical, clinical, and anthropometric variables after adjustment for age and sex, and χ^2^ test for categorical variables. Linear regression models were applied to capture the effect of polymorphisms on clinical phenotype variables, with age as the confounding covariate.

The significance level of the association test was set at 5%. Bonferroni correction was performed to correct multiple comparisons: for the genetic analyses, the significant *p*-value was set at 0.002 (0.05/5 × 2 × 3, where 5 represents the number of SNPs analyzed, 2 represents the sexes and 3 represents the number of comparisons). For the SNP association with biochemical parameters, the significant *p*-value was set at 0.0027 (0.05/18). Statistical analyses were carried out using SPSS software version 29.0 (SPSS, Inc., Chicago, IL, USA).

To evaluate if the detected effect of the polymorphisms on longevity may result in differential patterns of survival of the different relevant genotypes, univariate survival analysis was carried out using the Kaplan–Meier method and survival curves were compared using the log-rank test. Subjects alive after 10 years of follow-up were considered censored, and this time was used as the censoring date in the survival analyses. In addition, hazard ratios (HR) and 95% confidence intervals (95% CI) were estimated by using Cox proportional hazard models considering age and frailty as confounder variables.

## 3. Results

Table 1 reports the demographic, clinical, and biochemical characteristics of the study participants according to their frailty status.

As shown, the sample was divided into three sub-groups of frailty, namely non-frail (the cluster with subjects showing the best scores for the classification variables), indicated as G0, pre-frail (G1) and frail (the clusters with subjects showing the worst scores for the classification variables), indicated as G2. Frail subjects were older than pre-frail and non-frail and showed a higher percentage of females than males. As expected, frail subjects reported lower performance in cognitive and physical tests. Moreover, as for health-related parameters, significant differences were found among frailty groups for LDL and GGT values (*p* < 0.001); also, pre-frail subjects presented a higher number of co-morbidities, significantly different among the sub-groups (*p* < 0.001).

### 3.1. Association Between MT Polymorphisms and Frailty

Quality control procedures were performed on the seven initially genotyped SNPs, which included the exclusion of SNPs out of the Hardy–Weinberg equilibrium in controls (*p*-value < 0.05) or low genotyping success rates (<90%). Ultimately, two SNPs (rs11640851 in MT1A and rs10636 in MT2A) were excluded from analysis.

Allele frequencies and genotype distributions for all the SNPs were not significantly different between the frailty groups in the overall population.

**Figure 1:**
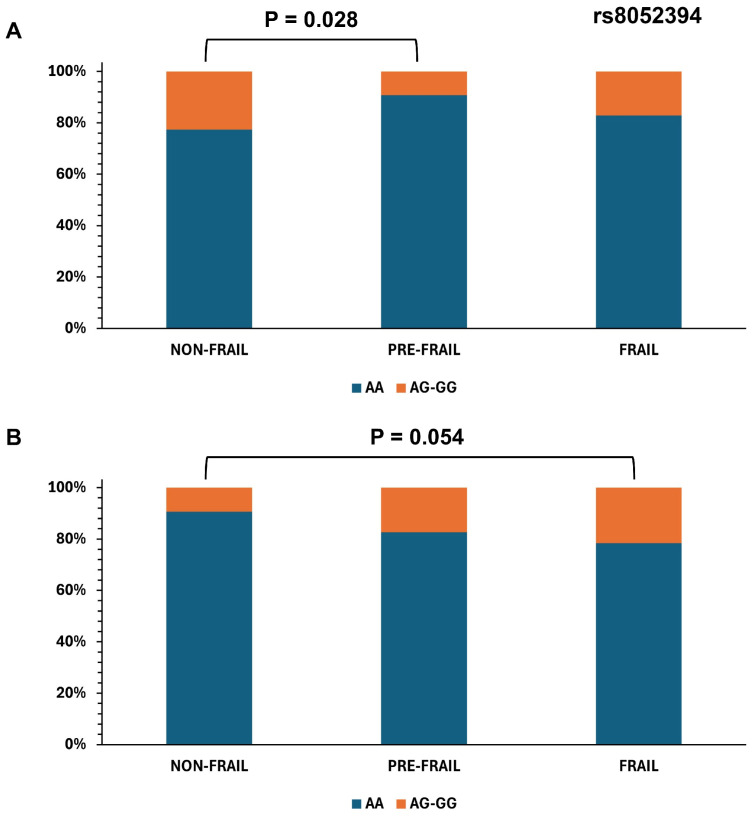
Distributions of genotypic frequencies at rs8052394-*MT1A* locus in the sample stratified for sex ((**A**), females; (**B**), males) and frailty groups.

To explore potential sex-specific associations, the analysis was conducted separately for females and males and a pairwise comparison of the groups (i.e., Go vs. G1; G1 vs. G2; G0 vs. G2) was performed. As shown in Figure 1 (panel A), in females the unadjusted analysis revealed a significant association with frailty for the variability of rs8052394-A/G at *MT1A* gene for the first comparison, with carriers of the minor allele showing a lower frequency in the pre-frail, with respect to the non-frail group (*p* = 0.028). In males (Figure 1, panel B), the unadjusted analysis reported a borderline significant difference (*p* = 0.054) for the variability of rs8052394-A/G at *MT1A* gene between the G0 and G2 status.

We next performed logistic regression analysis accounting for age. The adjusted analysis confirmed the association observed in females (OR = 0.345, 95% CI: 0.127–0.939; *p* = 0.037) (Table 2).

The association was also assessed using a multinomial regression model, which confirmed the previous result (OR = 0.343, 95% CI: 0.126–0.933; *p* = 0.036).

Although this association did not meet the Bonferroni correction threshold (p_corrected_ = 0.002) for multiple testing, the result is suggestive of a protective effect of the minor allele for the risk of transit from the non-frail to the pre-frail status.

We did not find any evidence of a nominally significant association between frailty and the other SNPs. Also, no association was found between the genetic variability and endophenotypes of frailty (MMSE, ADL and HGS, *p* > 0.05)

### 3.2. Association Between MT Polymorphisms and Health Status

To investigate whether the genetic association observed in females is related to specific health parameters, we analyzed the variability of the rs8052394-*MT1A* gene in the group of non-frail subjects with respect to the biochemical parameters listed in Table 1. We found that the variability at rs8052394-*MT1A* was associated with albumin levels, with G-carriers showing higher albumin levels than AA homozygotes (beta = 0.231; *p* =0.027), which does not survive Bonferroni’s correction (*p* = 0.0027). A trend of association was observed also for CRP levels with lower levels of CRP in G-carriers (beta = −1.563; *p* = 0.097). No significant results of association were reported for the other parameters.

### 3.3. Association with Survival

Given our finding of an association in females with the rs8052394 variant with the likelihood of passing from non-frail to pre-frail status in females and considering that this status preludes a deterioration of health condition which may translate to a shorter lifespan, we also assessed the effect of this polymorphism on the risk of all-cause mortality by using survival data at a 10-year follow-up. For this analysis, we corrected for age, sex and frailty status in the whole sample, and consequently for age and frailty in the sex-stratified analysis. No differences were found between carriers and non-carriers of the protective G-allele at rs8052394 in the whole sample (HR = 0.942; *p* = 0.778) and in the sample stratified by sex (HR = 0.990; *p* = 0.972; HR = 0.924; *p* = 0.807 in males). Thus, polymorphism does not influence survival.

## 4. Discussion

Metallothioneins (MTs) are a family of proteins crucial for maintaining cellular health. They are involved in a range of functions, including metal ion homeostasis, oxidative stress response, and inflammation [36]. Emerging evidence suggests a link between MTs and aging, as well as age-related diseases (see references in the Section 1). To our knowledge, this is the first study that examines the relationships between *MT* genes’ variability and frailty. We report that a variant of the *MT1A* gene, rs8052394-A/G, contributes to the risk of frailty in a sex-specific manner, without holding Bonferroni correction for multiple testing. Specifically, female carriers of the minor allele G were found to have a reduced likelihood of progressing from a non-frail to a pre-frail state, as compared to those homozygous for the major allele A.

MT1A, a major subtype of the MT1 isoform, is a multifunctional protein with roles in many different cellular processes. Under physiological conditions, it primarily binds zinc, making it a key player in protecting cells from oxidative stress. Zinc-sulfur clusters within MT1A are sensitive to changes in cellular redox conditions and oxidative stress, triggering the release of zinc to other proteins, including antioxidant enzymes [37]. This process helps to attenuate oxidative damage. In addition to zinc, MT1A can bind to other heavy metals like cadmium and copper, helping to maintain metal homeostasis and protect cells from toxicity. *MT1A* is also induced by various stimuli, including hormones, cytokines, and growth factors, and plays a role in several signal transduction pathways. These functions contribute to diverse biological processes, including inflammation, immunity, neuroprotection, and cell growth [38,39]. Given its involvement in these processes, MT1A is implicated in various pathological conditions, such as diabetes, tumors and neurological disorders [40,41,42]. The rs8052394 variant (also known as +1245 A/G *MT1A*) is a non-synonymous polymorphism located 152 nucleotides downstream of the start codon (ATG) in the *MT1A* gene. This SNP results in an amino acid change from lysine to arginine at position 51 (Lys51Arg). Rahman et al. (2022) investigated the structural consequences of the amino acid substitution caused by the rs8052394-G allele on the MT1A protein [30]. Their analysis indicated that this genetic variant negatively impacts protein’s conformation, compromising its stability. The rs8052394 has been implicated in various diseases, but its effects can vary across different disorders and populations, suggesting a pleiotropic role in disease-relevant conditions. The A allele of this variant has been associated with an increased risk of coronary artery disease (CAD) in diabetic patients [43]. Additionally, it has been linked to a higher risk of oral squamous cell carcinoma and potential alterations in zinc and copper homeostasis, which may influence molecules like p53 [44], and hepatocellular carcinoma [45]. On the other hand, the G allele of rs8052394 has been linked to an increased risk of type 2 diabetes mellitus (T2DM) in certain populations [46,47]. Furthermore, this allele has been associated with decreased superoxide dismutase (SOD) activity and antioxidant capacity [47], as well as elevated levels of advanced glycation end products (AGEs) and reactive oxygen species (ROS) [48].

In our analysis, the rs8052394-G allele appears to exert a protective effect for frailty risk in females. It is well established that sex significantly influences frailty risk. Despite greater resilience, women experience a higher incidence of frailty. This “sex-frailty paradox” is associated with factors such as longer lifespans, increased chronic disease burden, and higher disability rates. Several factors contribute to this paradox, including sex differences due to the presence of sex chromosomes, immune system function, metabolic differences, and hormonal changes [49]. Genetic and epigenetic factors are likely key drivers of sex-specific frailty, shaping the unique trajectories that men and women take towards this state. In this regard, research suggests that while frail males tend to experience issues related to muscle health, oxidative stress, and metabolic dysregulation, frail females often exhibit altered immune function and inflammation [50]. Based on this evidence, the variability of *MT1A* could influence frailty in women through an effect on these mechanisms. This effect may be mediated by the regulation of zinc availability, as MT1A not only acts as an intracellular reservoir of zinc, but its expression is also sensitive to zinc levels [51]. Zinc is an essential mineral, vital for numerous physiological processes, including maintenance of muscle function, immune health, cognitive function, and inflammatory response regulation, all of which are important in aging [52,53]. In the aged organism, the intracellular zinc homeostasis is altered, partly caused by nutritional deficiencies commonly seen in the elderly population, leading to lower overall zinc levels. Additionally, elevated levels of MT in older individuals further reduce intracellular zinc availability by sequestering zinc and limiting its release, leading to imbalances that can result in increased disease risk [54,55].

In this scenario, it is plausible to speculate that the positive effect of the G allele of rs8052394 on the frailty risk in women that we observed could be due to the increased instability of the MT1A protein caused by this allele. A less stable MT1A could result in diminished zinc-binding capacity and increased zinc release, thereby promoting intracellular zinc availability and potentiating inflammatory/immune signaling pathways. The association found in non-frail females between the G allele and increased albumin levels, with G allele carriers exhibiting higher levels than AA homozygotes, and the suggestive trend toward reduced CRP levels among G allele carriers, fits well with our suggestion that high levels of albumin and low levels of CRP are indicative of a low inflammatory status [56,57]. It should be noted that the associations we found were significantly related to the transition from the non-frail to pre-frail status. According to the literature, pre-frail status is the crucial shift from a state of complete recovery from stress to a condition of incomplete recovery, a prodromal state, which, if left untreated, may develop into frailty [58]. From this condition of “subclinical frailty”, the organism slips into the frailty conditions. Interestingly, women are often more vulnerable to developing a pre-frail status compared to men [59].

## 5. Conclusions

This study is the first to investigate the association between *MT* genetic variability and frailty. Our findings suggest a sex-specific link between *MT1A* variability and frailty status in women, with the polymorphism rs8052394 potentially influencing the transition from non-frail to pre-frail status. Additionally, associations with health parameters point to a possible effect of *MT1A* variability on the inflammatory status of female individuals.

We are aware that our work presents some limitations that deserve consideration. Firstly, the significance of our results does not withstand multiple comparison corrections, which may be due to our modest sample size, particularly after stratification into subgroups. As a result, the statistical power to detect small genetic effects is limited. Additionally, the lack of data on causes of death and the short follow-up period may explain the absence of significant findings, making it difficult to draw long-term conclusions about the effect of genetic variants with minor effects on survival. Nevertheless, these results will be valuable for future confirmatory research conducted with larger population samples. Secondly, the absence of reliable information on zinc levels in participants is a limitation, as this could provide further support for the proposed hypothesis. Thirdly, the complex nature of the frailty phenotype and the multifunctional role of metallothionein make it challenging to fully elucidate the underlying mechanisms of the observed associations.

Despite these limitations, the data presented here highlight the potential importance of MTs in the quality of aging. They also align with recent research on sex differences in frailty progression, contributing to the understanding of sex-specific genetic determinants of frailty, which could aid in the development of targeted strategies for the prevention and treatment of frailty. Therefore, this preliminary study could lay the groundwork for more in-depth future studies aimed at understanding the role of metallothioneins in the quality of aging in the human population.

## Figures and Tables

**Table 1:** Demographic, clinical, and biochemical characteristics of the study participants according to frailty status.

	Non-Rail (G0) N = 150	Pre-Frail (G1) N = 168	Frail (G2) N = 130	*p*-Values
Socio-demographic factors				
Sex % (male/female)	70.67/29.33	41.67/58.33	28.46/71.54	<0.001
Age	70.90 ± 6.11	74.13 ± 6.93	92.84 ± 6.62	<0.001 *^+≠^
Anthropometric parameters				
Body mass index, kg/m^2^	27.18 ± 3.68	27.63 ± 4.69	24.03 ± 4.44	NS
Height, m	165.13 ± 8.29	159.68 ± 8.67	151.4 ± 8.66	<0.001 ^+≠^
Cognitive and physical tests				
MMSE total score	27.65 ± 1.37	22.10 ± 3.47	14.50 ± 6.08	<0.001 ^+≠^
Activity of Daily Living	4.95 ± 0.21	4.87 ± 0.42	2.85 ± 2.01	<0.001 ^+≠^
Hand Grip Strenght	27.91 ± 9.24	21.08 ± 9.17	12.47 ± 5.84	<0.001 *^+^
Biochemical variables				
Fasting Glucose mg/dL	113.23 ± 35.9	112.82 ± 39.25	109.19 ± 43.31	NS
HbA1c %	5.73 ± 1.01	5.7 ± 0.97	5.3 ± 1.17	NS
Total cholesterol mg/dL	211.18 ± 46.47	205.88 ± 40.25	201.5 ± 43.12	NS
HDL mg/dL	56.59 ± 14.65	57.46 ± 15.07	55.02 ± 13.47	NS
LDL mg/dL	129.81 ± 39.19	120.98 ± 33.3	119.79 ± 34.04	<0.001 *
Triglyceride mg/dL	125.68 ± 73.53	137.11 ± 64.59	133.32 ± 65.02	NS
Albumin (g/dL)	4.01 ± 0.31	3.98 ± 0.29	3.75 ± 0.36	NS
Total protein	6.9 ± 0.38	7.32 ± 4.89	6.85 ± 0.61	NS
CRP (mg/dL)	5.25 ± 12.65	4.84 ± 5.64	6.6 ± 8.4	NS
FT3 (pg/mL)	3.43 ± 0.68	3.3 ± 0.66	2.8 ± 0.68	NS
FT4 (ng/dl)	1.36 ± 0.24	1.29 ± 0.27	1.27 ± 0.27	NS
TSH (μlU/mL)	2.28 ± 9.33	2.05 ± 6.42	1.39 ± 1.37	NS
AST (GOT) (IU/L)	25.61 ± 9.73	27.63 ± 32.57	25.42 ± 12.73	NS
ALT (GPT) (IU/L)	40.8 ± 12.53	45.29 ± 85.76	35.45 ± 21.39	NS
GGT (IU/L)	37.85 ± 21.39	37.5 ± 25.61	44.13 ± 47.36	<0.001 **^+≠^**
PT (seconds)	13.89 ± 2.2	14.34 ± 4.33	14.32 ± 2.52	NS
aPTT (seconds)	35.04 ± 4.45	37.66 ± 20.71	34.77 ± 4.36	NS
Fibrinogen mg/dL	361.99 ± 105.22	362.54 ± 72.95	387.22 ± 78.76	NS
Co-morbidities	3.3 ± 2.06	4.6 ± 2.36	2.47 ± 2.06	<0.001

Data are presented as (%) or mean ± standard error (compared by ANOVA adjusted for age and gender as covariates; post hoc analyses were performed using the Tukey tests) or percentage (compared using chi-square test). Abbreviations: MMSE: Mini Mental State Examination; HbA1c: Glycosylated hemoglobin; HDL: High Density Lipoprotein; LDL: Low Density Lipoprotein; CRP: C-Reactive Protein; FT3: free triiodothyronine; FT4: free thyroxine; TSH: thyroid stimulating hormone; ALT: alanine aminotransferase; AST: aspartate aminotransferase; GGT: gamma glutamyl transpeptidase; PT: Phrotrombin Time; aPTT: activated Partial Thromboplastin Time; *p*-value: NS (not significant); significant in comparison: * Non-Frail vs. Pre-Frail, ^+^ Non-Frail vs. Frail, ^≠^ Pre-frail vs. Frail.

**Table 2:** Results of the association test for MT SNPs, resulting from the logistic regression analyses.

	Go vs. G1			G1 vs. G2			G0 vs. G2		
SNP	O.R.	C.I.	p	O.R.	C.I.	p	O.R.	C.I.	p
Females									
rs11076161-MT1A	0.849	0.392–1.840	0.678	0.766	0.231–2.540	0.663	0.912	0.172–4.840	0.914
rs8052394-MT1A	0.345	0.127–0.939	0.037	1.360	0.260–7.160	0.713	0.969	0.112–8.390	0.977
rs964372-MT1B	0.825	0.393–1.730	0.610	0.859	0.294–2.510	0.780	1.850	0.394–8.660	0.436
rs1610216-MT2A	0.903	0.433–1.880	0.786	1.09	0.370–3.220	0.873	3.510	0.663–8.640	0.140
rs45570941-MT3	0.931	0.417–2.080	0.862	0.993	0.288–3.420	0.991	1.020	0.175–5.880	0.987
Males									
rs11076161-MT1A	1.720	0.883–3.350	0.111	0.512	0.077–3.430	0.490	0.378	0.037–3.850	0.411
rs8052394-MT1A	2.330	0.898–6.041	0.082	2.35	0.201–7.550	0.496	4.830	0.041–5.670	0.563
rs964372-MT1B	1.060	0.538–2.089	0.866	0.568	0.115–2.790	0.487	1.560	0.241–10.18	0.639
rs1610216-MT2A	0.917	0.480–1.751	0.793	0.506	0.01–2.570	0.411	1.570	0.224–11.00	0.650
rs45570941-MT3	1.380	0.679–2.797	0.375	0.297	0.04–2.200	0.235	0.816	0.065–10.25	0.875

Comparisons refer to the frailty classification of the sample in three sub-groups of frailty, namely non-frail (G0, pre-frail (G1) and frail (G2). Odd Ratio (O.R.), Confidence interval (C.I.) and relative *p*-values of the logistic regression analysis are reported.

## Data Availability

The original contributions presented in the study are included in the Supplementary Material. Further inquiries can be directed to the corresponding author.

## Supplementary Materials

The following supporting information can be downloaded at: https://www.mdpi.com/article/10.3390/genes16010015/s1, Table S1: Basic characteristics of the candidate genes and selected SNPs.
