# Supplementary material for: Metallothionein-1A (MT1A) Gene Variability May Play a Role in Female Frailty: A Preliminary Study
Source: Genes (Basel). 2024 Dec 26;16(1):15. doi: 10.3390/genes16010015 (PMC11765288; doi:10.3390/genes16010015)
Supplement: Supplementary file 1 [file genes-16-00015-s001.zip › Table S1.pdf]

Table S1. Basic characteristics of the candidate genes and selected SNPs.

Gene	SNP	Chr: Position **(GRCh38.p14)**	Restriction enzyme	Major/Minor allele	Mutation Type	MAF Ensemble (TSI)	MAF Control sample
MT1A	rs11076161	16: 56639236	ApoI	G/A	intronic variant	0.26	0.28
MT1A	rs8052394	16: 56639916	PstI	A/G	Missense variant	0.15	0.07
MT1A	*rs11640851*[a]	*16: 56639315*	*MnlI*	*A/C*	*Missense variant*	*0.38*	*Low call rate*
MT1B	rs964372	16: 56652118	MscI	C/G	3' UTR variant	0.16	0.33
MT2A	rs1610216	16: 56608372	SmaI	A/G	TF binding site	0.18	0.28
MT2A	*rs10636*[b]	*16: 56609431*	*MaeIII*	*C/G*	*3′ UTR variant*	*0.20*	*Not HWE*
MT3	rs45570941	16: 56590312	BamHI	G/C	intronic variant	0.12	0.18

NB: Minor Allele Frequencies are relative to the Tuscany population (Eur) in Ensembl.org. In italics, the SNPs excluded from the analysis because of a low call rate (a) or HWE departure (b) were reported
